# Using Self-Generated Cues to Facilitate Recall: A Narrative Review

**DOI:** 10.3389/fpsyg.2017.01830

**Published:** 2017-10-27

**Authors:** Rebecca L. Wheeler, Fiona Gabbert

**Affiliations:** Department of Psychology, Goldsmiths, University of London, London, United Kingdom

**Keywords:** retrieval cue, encoding-specificity, spreading activation, cue distinctivness, cue generation, self-generated cue, Mental Reinstatement of Context, encoding-retrieval match

## Abstract

We draw upon the Associative Network model of memory, as well as the principles of encoding-retrieval specificity, and cue distinctiveness, to argue that self-generated cue mnemonics offer an intuitive means of facilitating reliable recall of personally experienced events. The use of a self-generated cue mnemonic allows for the spreading activation nature of memory, whilst also presenting an opportunity to capitalize upon cue distinctiveness. Here, we present the theoretical rationale behind the use of this technique, and highlight the distinction between a self-generated cue and a self-referent cue in autobiographical memory research. We contrast this mnemonic with a similar retrieval technique, Mental Reinstatement of Context, which is recognized as the most effective mnemonic component of the Cognitive Interview. Mental Reinstatement of Context is based upon the principle of encoding-retrieval specificity, whereby the overlap between encoded information and retrieval cue predicts the likelihood of accurate recall. However, it does not incorporate the potential additional benefit of self-generated retrieval cues.

## Introduction

Being able to reliably recall a personally experienced event is sometimes of critical importance. A good example is when an eyewitness is required during a police investigation to give a complete and accurate account of criminal activity witnessed. In a more everyday context, the recall of personally experienced events can function as a means to understand ourselves and others in the world around us. Through recalling personal memories we can identify who we are as an individual consistent over time, learn from the past, solve current problems, and plan for the future. We can also strengthen social ties and build and maintain intimacy in our relationships through the sharing of stories about past events (Fivush, 2008; Harris et al., 2014).

Successful recall of information from memory is often dependent upon the provision of retrieval cues (see Tulving, 1974 for discussion). Retrieval cues are aspects of an individual’s physical and cognitive environment which aid the recall process; they can be explicitly provided at recall, self-generated, or encountered more incidentally through the retrieval context (Pansky et al., 2005). Given the potential importance of accurate recall of personally experienced events outlined above, it is unsurprising that numerous mnemonic techniques have been developed to facilitate this process. The most successful of these build upon established principles of memory, such as the idea that encoding information leaves behind a memory trace comprised of multiple pieces of related information. This means that effective retrieval cues are those which contain a large amount of overlap with encoded information, and that different retrieval cues may facilitate the recall of different items of information (Geiselman et al., 1986).

In the discussion that follows we outline the qualities necessary for a retrieval cue to be effective, and based upon the extant literature, argue that self-generated retrieval cues represent a unique opportunity to maximize each of these qualities. We contrast use of self-generated cues with established context reinstatement techniques, in particular Mental Reinstatement of Context, found principally within the eyewitness domain. Based upon this discussion, we argue that the theory underpinning Mental Reinstatement of Context also supports the effectiveness of self-generated cue mnemonics, and that self-generated cues offer an additional opportunity to capitalize upon the benefit of cue distinctiveness. We close by outlining three memory principles underlying each of these mnemonic techniques: spreading activation, encoding-specificity, and cue distinctiveness. Our aim throughout this review is to consider how existing memory theories might contribute to the beneficial effect of self-generated cues on recall, as demonstrated by the empirical studies outlined, and not to consider alternative explanations of these findings.

## Discussion

### Episodic Memory

The recall of personally experienced events falls within the domain of episodic memory. Episodic memory, first proposed as a memory system by Tulving (1972), consists of highly detailed sensory information about recent experience. It principally involves recalling the what happened, where, and when of events. As such, episodic memory deals more with personal experience than with general facts about the world and ourselves. It is the ‘personally experienced’ aspect of episodic memory that distinguishes these memories from semantic memories for more general facts (Tulving, 2001). This concept has been revised by Conway and colleagues to define episodic memory as a system containing highly event-specific, sensory-perceptual details of a recently experienced event. These events usually cover a relatively short-time span, often lasting just minutes or hours (Conway, 2001). It is the high levels of sensory-perceptual detail incorporated into episodic memories that make the re-experiencing of previous events possible through ‘mental time travel,’ something Tulving argues is likely to be unique to humans (Tulving, 2001, 2002). Tulving (2002) suggests that the episodic memory system is relatively early-deteriorating, and Conway (2001) argues that episodic memories persist on a longer-term basis only when incorporated into autobiographical memory structures (indeed Conway argues that autobiographical memory structures typically consist of one general event, alongside at least one episodic memory). Autobiographical memory, in contrast to the shorter-term, high event specificity of episodic memory, can be taken to be a system of long-term memory containing three levels of specificity: lifetime periods, general events, and event-specific knowledge. It is also generally considered that the self is of central importance to autobiographical memory (Conway and Pleydell-Pearce, 2000). Here, we refer to episodic memory in line with Tulving’s (1985) suggestion of episodic memory as a specialized subcategory of memory relating to the conscious recall of personally experienced events. In this sense, episodic memory is both a particular type of encoded information, and a particular type of recollective experience (Tulving, 2002).

### Effective Retrieval Cues

A number of key qualities have been suggested as necessary for a retrieval cue to effectively support recall. Good quality retrieval cues often have: (i) constructability (cues generated at encoding can be reliably reproduced at recall); (ii) consistency between encoding and retrieval within a given context (i.e., an effective retrieval cue should be compatible with the memory trace created during encoding and show high cue-target match); (iii) strong associations with the target and the ability to be easily associated with newly learned information; and (iv) bidirectionality of association (the cue recalling target information, and target information recalling the cue). It is also important that retrieval cues are distinctive or discriminable. That is, it should be possible to distinguish cues from one another, and to differentiate the target memories associated with each. If retrieval cues are not recognized as being distinct from one another, then cues are likely to become associated with more information, which in turn reduces the effectiveness of the cue in prompting the recall of target information. This is known as cue overload (Watkins and Watkins, 1975), which leads to slower less accurate recall as a result of a cue (node) containing too many associative links (the fan effect; Anderson, 1983a). In addition, fuzzy trace theory (e.g., Brainerd et al., 1995) suggests that multiple traces are encoded within memory for a single event. In other words, separate memory traces are created which contain either general information about an event (gist traces) or exact details of the same event (verbatim traces). It has been suggested that gist traces are likely to be activated by a wider range of retrieval cues than verbatim traces (Tuckey and Brewer, 2003). This means that more distinct retrieval cues are necessary to access detailed target information (Bellezza and Hoyt, 1992; Tullis and Benjamin, 2015a).

### Self-Generated Cues

The self-generation of cues to prompt recall of information at a later date is a relatively natural process; for example, individuals regularly create file names to cue themselves as to the contents, create slides to prompt themselves as to presentation content, or take notes on important information to allow detailed recall in the future (Tullis and Benjamin, 2015b). Generally, it can be expected that individuals should be effective at generating cues to prompt their own future recall. When generating cues ourselves we are able to rely upon rich, unique, personal knowledge to produce cues which are often distinctive, highly associated with the target, and consistent between encoding and retrieval (and therefore stable over time). Research has demonstrated that individuals do not consistently favor any one of these principles over the others when self-generating retrieval cues; instead, they utilize these characteristics flexibly to fit with the current task demands (Tullis and Benjamin, 2015a). For example, when learners are provided with information about the similarity of competing targets (they were made aware that targets were similar to one another) prior to generating their cues, they focused more on distinguishing between the targets through maximizing cue distinctiveness, and so improved their performance on a recall task (Tullis and Benjamin, 2015a).

#### Defining a Self-Generated Cue

Research has suggested that the most effective self-generated cues are likely to have been developed with the explicit purpose of cueing later retrieval. This helps individuals to make deliberate choices distinguishing the target from other items stored within memory, rather than merely describing the properties of the target (Tullis and Benjamin, 2015a). In this way, developing self-generated cues can be considered as an active process, resulting in cues which uniquely and functionally represent the critical properties of the target memory (Mäntylä and Nilsson, 1983). For example, when learners were told directly that the cues they generated would be used to guide a future retrieval attempt (mnemonic cues), their cues tended to include more idiosyncratic knowledge and personal experience, were more distinctive, and associated to fewer potential targets, and so facilitated greater levels of recall than cues generated to simply describe the target (Tullis and Benjamin, 2015a). Self-generated cues are likely to include idiosyncratic details based upon the personal context of encoding. They are also likely to make particular use of distinctive aspects of the information to be encoded to distinguish the representation of the target memory from others already stored in memory (Mäntylä, 1986).

As far as we are aware there is no widely agreed definition of a self-generated cue. Here, we refine the definition of a self-generated retrieval cue to mean any detail salient to the individual, and actively generated by the individual themselves, which serves to facilitate more complete retrieval of a target memory, and as such represents the critical properties of the target memory. In defining a self-generated cue, it is also important to distinguish our interpretation of a self-generated cue from other similarly named concepts within the domain of memory research. For example, from the related concept of the generation effect, as well as from self-referent cues commonly found in the autobiographical memory literature. Each of these is treated individually below.

##### The generation effect or elaborative processing

It has been suggested that information is better recalled when it has been actively and effortfully processed, rather than passively received. This can be considered as a *necessary* but not *sufficient* prerequisite for unique encoding (Slamecka and Graf, 1978; Mäntylä and Nilsson, 1983). Production of unique cues at the encoding stage encourages enhanced encoding of target material. One means of inducing more active unique encoding is to have participants generate the stimuli to-be-recalled for themselves. For example, participants might be given a word with some letters replaced with blanks. This is often presented alongside a strong semantic cue (e.g., *fruit*: a p _ l _). Learners are asked to complete the word, and then to encode this word for later recall (Schmidt, 1991; Laffan et al., 2010). Self-generated stimuli are more accurately recalled than stimuli passively encoded under the same conditions, and this effect persists over a longer retention period. This effect (known as the *generation effect*) holds constant across a range of measures such as cued and uncued recognition, free recall and cued recall, and confidence ratings (Slamecka and Graf, 1978; Mäntylä and Nilsson, 1983; Laffan et al., 2010).

The generation effect can be considered as representing the impact of deeper, semantic, more distinctive encoding strategies (Derwinger et al., 2005). While this potentially works on some of the same principles as our definition of self-generated cues, these two processes are subtly different. In essence, it seems that when a generation effect approach is taken, learners are generally trying to generate the encoding material. In contrast, a self-generated cue in our context is one that is generated by the individual (and so can be as idiosyncratic as necessary) to prompt the recall of encoded material, but does *not* necessarily consist of the target material itself. It is worth noting that some research has found that the generation effect improves memory for target items, but can lead to a reduction in memory for contextual details (Mulligan, 2004; Mulligan et al., 2006). It is not yet known whether self-generated cues might also fail to enhance memory in all contexts.

##### Self-referent cues

References to ‘self-referent cues,’ ‘self-relevant cues,’ or ‘personally relevant cues’ are not uncommon in the autobiographical memory literature. It has been suggested that there is a strong relationship between the self and memory, and that in particular the self-referencing of autobiographical memories distinguishes them from other types of memory (Conway and Pleydell-Pearce, 2000). In addition, it has been suggested that memory is, at least in part, organized around the concept of the self (see for example Greenwald and Banaji, 1989; Symons and Johnson, 1997). A self-referent cue generally involves processing information in reference to the self. In the simplest terms, this means thinking about oneself during the encoding process (Turk et al., 2015). In doing so the individual associates a piece of to-be-remembered information with a self-relevant item (as in Greenwald and Banaji, 1989). This has been shown to have broader implications for recall, as well as impacting achievment in educational contexts (as in Turk et al., 2015). However, this is somewhat different from the definition of a self-generated cue to (non-autobiographical) retrieval we outlined above. The main distinction being that self-generated cues reflect those that represent critical properties of a target memory, while self-referent cues are those that act as a cue relating to an aspect of the self.

#### The Benefit of Self-Generated Cues Over Cues Generated by, or for, Others

It is well-established that strong cue-target relationships, cue distinctiveness, and compatibility between encoding and retrieval are necessary to maximize the effectiveness of a retrieval cue. It is reasonable to assume then that if we are able to capitalize upon each of these principles, then recall performance will be further improved. If this is the case, then allowing individuals to generate their own retrieval cues represents our best opportunity to utilize cues that are unique, and include a high level of cue-target match. Indeed, some researchers have already argued that the high levels of recall demonstrated when the target information shares a unique relationship with the cue become more striking when the cue is self-generated (Hunt and Smith, 1996). This is not altogether surprising; if effective retrieval cues are both distinctive and compatible with the encoding experience, then it follows naturally that cues are more effective when they are self-generated than other-generated. The ‘tester’ cannot know what information was most salient to the learner at the time of encoding, nor can they anticipate which aspects of that information are most distinctive to the learner (Mäntylä, 1986). As a result, other-generated cues (i.e., cues that are formulated by someone other than the individual themselves) rely heavily upon more general, semantic, gist-based aspects of the target information, rather than the more specific idiosyncratic episodic details incorporated into self-generated cues. In this sense, other-generated cues can be considered to rely primarily upon associative strength (between cue and target), without the additional benefit of cue distinctiveness and encoding-retrieval match offered by self-generated cues. In support of this, Tullis (2013) highlights that when learners recalled an incorrect target, this response appeared to be driven by the associative strength between the cue and the incorrect response. This suggests that when learners are unable to access specific episodic details for a cue they resort to a ‘best guess’ based upon associates of the cue provided to them. In other words, when specific episodic details are unavailable, learners fall back upon more general semantic knowledge. This suggests that strong cue-target associations (favored by spreading activation theories of memory) are the backup route to recall, when cue-target overlap and cue distinctiveness fail.

It has been argued that research into self-generated cues makes an important contribution *beyond* the understanding of cue distinctiveness. For example, in examining the use of self-generated cues, we are able to move beyond understanding encoding as the perception and comprehension of an item, to viewing this process as an additional source of potential retrieval cues (Hunt and Smith, 1996). This argument was based primarily around the extraordinary findings of Mäntylä and Nilsson (1988) who showed that given distinctive self-generated verbal cues and a consistent encoding-retrieval environment, recall of unrelated verbal targets is consistently of a high level, even with a long retention interval. This advantage is specific to the producer of the cue, with the cue itself failing to function effectively as a prompt for another individual’s recall. In effect, even where two individuals have encoded the same information, they are likely to produce unique retrieval cues, and so benefit exceptionally well from their own cues.

The retrieval benefit of self-generated cues over other-generated cues has been suggested as being linked to the generation process (e.g., through encouraging more active processing of the target memory). However, the research outlined above suggests that this benefit is the result of both the generation *process*, and the generation *context*. The potentially idiosyncratic nature of self-generated cues means that one individual’s cues that are given to another individual at test would be unlikely to benefit their performance, even if the same information had been presented at encoding. Despite this, individuals do frequently generate cues to benefit others in naturalistic settings. For example, we might consider how best to prompt an employee to complete a task, or cue one another’s memories for shared events when reminiscing with friends (Tullis and Benjamin, 2015b). It is then interesting to examine how asking individuals to generate cues specifically for use *by others* impacts upon the types of cues generated, and the effectiveness of these cues at test. During one such study participants generated cues for themselves and cues for others. At recall, they received another person’s cues (this could be a friend or stranger), but never their own self-generated cues. Results suggest friends are able to cue each other more effectively than strangers. However, performance overall improved when participants were provided with cues generated with the knowledge that the cue would be used to support someone else’s recall (Andersson and Ronnberg, 1997, Experiment 2).

Tullis and Benjamin (2015b) examined how the quality of a retrieval cue changed when it was generated for use by others rather than use by the self. Participants each generated two cues for each of 60 words. These cues were to be used to support their own later recall attempt, or to aid another learner in recalling the items on the wordlist. The stimulus words were selected as having relevance to the life of college students, and so were considered to offer opportunities for the use of cues based on personal experience. Cues presented at recall were either self or other-generated, and were intended for use by either the self or another individual. In general, cues generated for the self were consistently more idiosyncratic, and so less beneficial when presented to another learner. Consequently, performance was better when participants received an other-generated cue meant for another individual, than an other-generated cue meant for the self. In addition, self-generated cues intended for another individual were no longer as effective in facilitating the originator’s recall performance. Although this difference did not reach significance, this does suggest that the benefit of self-generation of the cue is removed when self-generated cues are intended for use by others. This is perhaps as a result of the reliance on more semantic cue-target associations, rather than distinctive, and often idiosyncratic details, of the encoding experience. It can therefore be assumed that the benefit of self-generated cues lies in the inclusion of personal experience and idiosyncratic knowledge to create a distinctive and meaningful cue.

#### Empirical Tests of Self-Generated Cue Mnemonics

Mäntylä and colleagues were among the first to note the benefit of self-generated cues on recall. Mäntylä and Nilsson (1983) were able to demonstrate strikingly high levels of recall (around 96% of a 30-word list), but only when participants were able to self-generate retrieval cues, and when these same retrieval cues were presented at test. These extraordinarily high levels of recall have been replicated in other contexts. For example, when participants were able to generate three cues at encoding, and then received these cues during an immediate recall test they recalled around 90% of up to 600 words. Performance levels declined slightly when only one self-generated cue was presented at test (to around 50–60%), but self-generated cues consistently resulted in high levels of performance. When other-generated cues were presented performance was particularly low (around 5% given one cue, rising to 17% when three cues were presented; Mäntylä, 1986). This suggests that the benefit of self-generated cues lie with the inclusion of idiosyncratic details within the cues, resulting in a unique cue which overlaps with few targets. It is then unsurprising, in terms of the encoding-specificity principle of memory, that these cues were only beneficial when they were self-generated (Hunt and Smith, 1996).

The high levels of performance demonstrated by Mäntylä and Nilsson (1983) and Mäntylä (1986) did however decline considerably as the retention interval increased. This decline was suggested as being the result of a decrease in the compatibility of the encoding and retrieval context, stipulated as a requirement of effective recall by the encoding-specificity principle of memory (Mäntylä, 1986). If this is the case then it is possible that that retrieval is impaired because the meaning of a cue is interpreted differently at encoding than at recall, and so consistent use of cues could help to maintain levels of performance. Essentially, reducing *within participant* cue variability for the same target item should reduce the decline in performance. Mäntylä and Nilsson (1988) asked participants to focus in particular on distinctive properties of the target when generating a cue in an attempt to reduce the intrasubject variance (and so make it more likely that the exact same cue will be produced on more than one occasion). They showed that when cues are generated with distinctive features in mind, then the decline in performance over time is much smaller (in comparison to a group who generated their own cues according to personal experience as an appropriate description of the target word) than has been previously suggested (e.g., Mäntylä, 1986). This effect persists throughout a retention interval of up to 6 weeks. This suggests that asking learners to focus specifically on distinctive aspects of the to-be-recalled information during encoding results in self-generated cues which maximize distinctiveness in a way that is unaffected by changes in context (reduced levels of encoding-retrieval match), and in turn ensures that levels of performance are maintained over time (Mäntylä and Nilsson, 1988).

Self-generated cues have also been shown to be effective in recalling more complex stimuli. For example, recall of paragraphs of text has been showed to improve with use of self-generated cues. van Dam et al. (1987) asked participants to study 20 standalone paragraphs in a factual narrative. Recall of the contents of each paragraph was more complete when participants were able to first generate a list of keywords (from memory) that they felt represented the content of each paragraph (i.e., the generated keywords did not have to be present in the paragraph). Interestingly, this was only effective when keyword generation took place *before* the first full recall attempt. When an initial recall of the paragraph contents was attempted, and then the keywords were generated to supplement this attempt, self-generated cues had no impact on the amount recalled.

Furthermore, research has suggested that there is a potential benefit of self-generated cues for those experiencing the beginnings of cognitive decline. For example, use of self-generated cues has been shown to facilitate the recall of a word list in both young adults (aged 20–39) and older adults (aged 70–89). Learners generated cues that were either semantic or phonetic (rhyming) dependent upon the instructions given. A benefit of self-generated cues was shown regardless of the level of processing at which the cue was generated. However, the benefit was more pronounced for older adults, and in particular self-generated semantic cues greatly reduced age-related differences in performance (Sauzéon et al., 2013). The fact that self-generated cues may benefit older adults more than younger adults is particularly striking, and further distinguishes self-generated cues from self-referent cues. For example, while both younger and older adults have been shown to benefit from encoding items to be recalled with reference to the self, research has suggested that older adults benefit less from self-referent processing than younger adults. In particular, it has been suggested that the effectiveness of self-referent encoding varies dependent upon the availability of cognitive resources, and that older adults are more limited in their ability to use this technique flexibly (Gutchess et al., 2007).

In addition, training in the use of a mnemonic, whether this was an established mnemonic or a self-generated strategy, has been shown to improve four-digit number recall of older adults. Older adults were trained using a number-consonant mnemonic (whereby a series of number-consonant pairs are memorized, and a word-phrase generation technique used to memorize number strings) or asked to use a systematic approach during practice sessions to develop an effective strategy for recalling the target digit-strings. The self-generated strategy group were asked to monitor their encoding processes and to make a note of the strategy they adopted to memorize each four-digit number string. For example, in attempting to memorize 2468 participants might enter “my birth year (24), my wife’s age (68),” “digit sequence beginning at 2 and adding 2,” etc. If participants were unable to think of a specific strategy they might report “repeated the numbers,” etc. In this way the participants retrieval strategies, and the reporting of these strategies, was not constrained in any way. Both trained groups outperformed a control (who received no training or practice time) at pre-test and post-test, both with and without cognitive support (cognitive support consisted of the generation of a word cue to prompt recall). Between the two training groups, the mnemonic group showed an improvement in performance from pre-test to post-test, and this improvement was magnified when post-test support was provided. In contrast, the self-generated strategy group showed a (non-significant) improvement from pre-test to post-test without support. This reached significance when post-test support was provided. The fact that both groups showed broadly similar levels of improvement from pre- to post-test is particularly striking when it is considered that the self-generated strategy group received slightly less training than the mnemonic strategy group (Derwinger et al., 2003). The gains in performance made by both the trained groups were also shown to persist after an 8-month delay (Derwinger et al., 2005). This gain persisted for the self-generated strategy group even when cognitive support was removed (the trained mnemonic group in contrast showed a decline in performance at this stage). These findings suggest that cognitive support is less necessary for the benefit of self-generated strategies to be maintained, in comparison to a more cognitively demanding mnemonic technique (Derwinger et al., 2005).

Although self-generated cues and self-generated mnemonic strategies have been used successfully by older adults, it is important to note that this finding is not as clear cut as might first appear. For example, Mäntylä and Bäckman (1990, Experiment 2) demonstrated that when participants were asked to recall a target word in response to presentation of a cue word self-generated 3 weeks prior, younger adults outperformed older adults. Mäntylä and Bäckman argue that these results reflect an age-related increase in encoding variability. For example, when both younger and older adults were asked to generate properties for target words in two sessions up to 3 weeks apart (with the instruction in the second session to generate properties describing their current interpretation of the target word, rather than trying to recall the descriptions generated in the first session), older adults were less consistent in the properties generated. Older adults also tended to rely on more generic properties, rather than utilizing more distinctive idiosyncratic properties (Mäntylä and Bäckman, 1990, Experiment 1). They suggest that this increase in age-related encoding variability is likely to contribute to the decline in episodic recall performance. Despite this, the potential benefit of self-generated cues in facilitating recall of both younger and older adults is something which merits further research.

### Context as a Retrieval Cue

Retrieval cues can also come from the context of an event. The contextual dependence of memory and the benefit that physical or mental reinstatement of encoding conditions at retrieval can have upon recall has long been established in laboratory research (see for example, Smith, 1979). The relationship between memory and context is a natural extension of the encoding-specificity principle of memory (Tulving and Thomson, 1973). In addition, the provision of contextual cues may enhance the completeness of recall through facilitating the spread of activation from accessible items to those not initially accessible (Hershkowitz et al., 2002).

One of the most established and frequently tested context reinstatement techniques is the Mental Reinstatement of Context. This is one of the cognitive mnemonic techniques incorporated into the Cognitive Interview (developed by Fisher et al., 1984). Mental Reinstatement of Context describes the process of guiding the individual to reconstruct an internal representation of the physical context of an event. This generally includes instructions to “reinstate in your mind the context surrounding the event” through considering the layout of the scene, the weather, the people and objects that were nearby, and so on. It is also considers the personal context of the event, through attempting to recall thoughts, feelings, and reactions to the event to-be-recalled (Geiselman et al., 1985). This technique is frequently used within laboratory studies on eyewitness memory. A recent meta-analysis suggested that 100% of the studies conducted using the CI and its variants over the preceding 25 years had incorporated MRC instructions (Memon et al., 2010). It is also noted as being a highly effective recall technique. For example, provision of physical cues from encoding and encouraging mental reinstatement of the context of the event has been suggested to increase the accuracy of identifications in an eyewitness context (Krafka and Penrod, 1985). This process has been shown to result in an increase in the level of detail (although not necessarily the amount of detail) provided in real-world accounts (e.g., Hershkowitz et al., 2002).

#### The Benefit of Self-Generated Cues Over Context Reinstatement

It has been suggested that the benefits of context-based cues become more apparent only when more effective cues are unavailable, suggesting that the benefit of context-based mnemonic approaches can be overshadowed if individuals are able to provide their own cues (Pansky et al., 2005). One potential means of reinstating context whilst also encouraging the use of an individual’s own cues is the Sketch Mental Reinstatement of Context. Developed by Dando et al. (2009, 2011) this technique allows trained interviewers to guide individuals toward using their own contextual cues when recalling a complex event. When using this technique, the witness sketches details of the event to be recalled, describing these aloud as they do so. Use of the Sketch Mental Reinstatement of Context has been suggested as comparable to the standard Mental Reinstatement of Context procedure in terms of both accurate information elicited and overall accuracy. The additional benefit of the Sketch Mental Reinstatement of Context is that it introduces self-generated contextual cues which are likely to be more salient (and so more effective) than contextual cues provided by an interviewer (for example through the standard MRC procedure).

However, even where context reinstatement techniques can be combined with self-generated retrieval cues, there remains problems with the application of these techniques. Context reinstatement techniques such as Mental Reinstatement of Context can be both difficult and time-consuming to implement effectively. For example, trained interviewers report finding Mental Reinstatement of Context (and other Cognitive Interview techniques) cognitively demanding, requiring flexibility, and difficult to incorporate in real world settings (Kebbell et al., 1999; Brown et al., 2008). It should be noted here that the Sketch Mental Reinstatement of Context technique has been suggested to reduce some of these demands, but more research is needed before this can be stated conclusively.

In contrast, the limited work that has investigated the use of self-generated cues in an applied context suggests that they might be preferable to techniques which require greater levels of training. As Derwinger et al. (2005) suggest the ease of use and personal compatibility inherent in self-generated strategies may mean that they are relatively easily incorporated into everyday routine, thus providing practice effects over time. The self-generated cue research described thus far has some applied relevance, but still relies primarily upon fairly artificial stimuli and artificial means of self-generated cue production. The work outlined in the following section begins to take steps to move the use of self-generated cues into a more ecologically valid domain.

When faced with a complex event, particularly one rich in temporal details or involving multiple actors, accurate recall of information becomes a more cognitively demanding task. Interviewee-led cueing methods have begun to appear in an eyewitness domain, and these techniques show undoubtable promise. For example, Hope et al. (2013) demonstrated that use of the timeline technique facilitated retrieval in an eyewitness testimony context. When using this technique individuals are able to delineate a complex event into key stages by placing person description cards and action cards on a physical cardboard timeline. This allows the interviewee to recall the individuals, actions, and sequences involved in a complex event in a witness-compatible manner (e.g., by beginning at the most salient point of the event). Use of this technique has been shown to facilitate the retrieval of more details than a free recall account alone, with no cost to accuracy. This benefit persists even after a 2-week delay. Furthermore, use of multiple mnemonics, including self-generated cues, during an interview about repeated events (in this case family gatherings) facilitated witness recall, even when the witness judged that they had recalled as much as they were able (and after repeated attempts to keep trying yielded no more information). Results showed an increase in recall of around 70% when using a combination of seven distinct mnemonics than when recalling unaided (Leins et al., 2014). Taken together these findings suggest that self-generated cues may be an intuitive means of facilitating recall in everyday settings.

### Theoretical Underpinnings of Self-Generated Cue Mnemonics

The research outlined thus far suggests a clear benefit of the use of self-generated cues on retrieval. We now address the theory underlying this approach. There are three key principles of memory which contribute to explaining the effectiveness of self-generated cues: the spreading activation theory of memory, the encoding-specificity principle of memory, and cue distinctiveness. We outline each of these in turn in the sections that follow, and speculate on how these principles of memory relate to the success of self-generated cues in aiding retrieval.

#### Spreading Activation Theory of Memory

In attempting to recall information from episodic memory we have to access long-term memory, a relatively slow process in comparison to other human information processing systems (Anderson, 1983a). Spreading activation models view information in long-term memory as being represented by a network of associated concepts. The assumption is then that it is possible to recall a given item from memory by recalling other information associated with the target. This is made possible through the process of activation spreading through the network (Anderson, 1983a; Crestani, 1997).

Memory is generally viewed as a network of interlinked nodes (as in Collins and Loftus, 1975; Anderson, 1983b). Within these networks, units of memory are conceptualized as cognitive units, made up of a node and its associated elements (or key properties of the node). Cognitive units make up the essential units of encoding and retrieval. During encoding, a cognitive unit is formed via a copy in working memory, which is later transferred as a more permanent long-term memory trace (Anderson, 1983b). Associative networks are formed of generic nodes, representing concepts or categories and knowledge about the category member, and episodic nodes, representing specific instances of generic nodes, connected by associative links (Tuckey and Brewer, 2003). There has been some debate around whether cognitive units are limited or unconstrained in terms of the number of linked elements they are able to contain. Irrespective of this, it is likely that memory networks represent a complex structure of links between concepts and associated properties (see Collins and Loftus, 1975; Anderson, 1983b, for examples of opposing views on this issue).

Spreading activation models generally assume that when information is encoded in memory it is also incorporated into a semantic network. In other words, information can be considered as being organized around semantic similarities. If this is the case, then the extent to which any one concept primes activation of another is a function of the number of connections between the two concepts. In other words, as activation spreads between semantically related memories during a recall attempt, the recall of one item often primes the recall of other semantically related items and so on (for further discussion of this assumption and the underlying experimental data see, Collins and Loftus, 1975).

Further support for the assumption of semantic organization of memory networks is shown through the use of category clustering recall techniques. Paulo et al. (2016) examined whether recall of a complex eyewitness event could be improved by asking participants to recall the target event in terms of the person, object, action, and location details of the event. Their results suggest that this category clustering is an effective mnemonic technique. Paulo et al. (2016) suggest that according to Collins and Loftus (1975) spreading activation theory of semantic processing, a key benefit of recalling via semantic (or category) clusters is that this approach gradually allows activation within the network to reach a level which triggers other semantically related information which may not otherwise have been activated and recalled.

**FIGURE 1:**
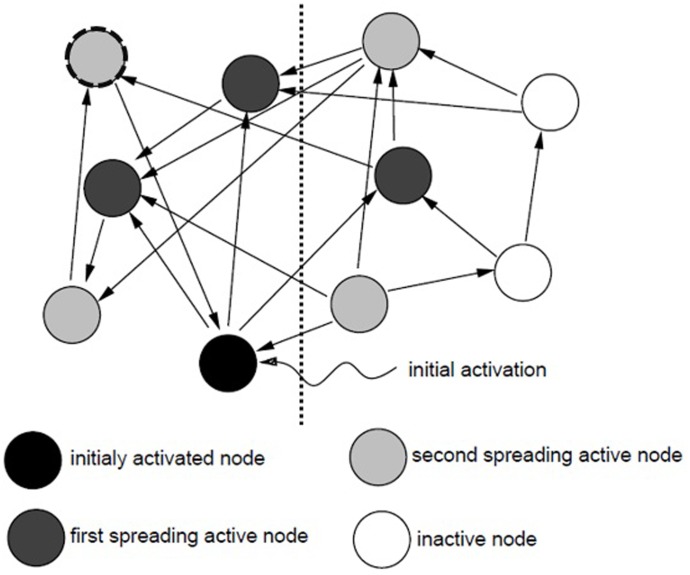
The spread of activation through a memory network (adapted from Crestani, 1997).

Spreading activation models of memory all generally view a memory search as the process of spreading activation from concept nodes along associative links throughout a semantic network until a threshold is reached (Collins and Loftus, 1975). The original spreading activation theory was proposed by Quillian (1962, 1967) who attempted to develop computer simulations of human memory search (see also developments by Collins and Loftus, 1975; Anderson, 1983b). It is generally accepted that a memory cue (sometimes termed a memory probe) triggers a memory search beginning at the node or nodes originally activated by the cue. The activation then spreads to all nodes connected to the initial node, and then to all nodes linked to these first tier activated nodes, and so on (Collins and Loftus, 1975). As activation spreads throughout the network information associated with the sources of activation becomes available (Anderson and Pirolli, 1984). This process is shown in **Figure 1** below. In this example, the cue triggers activation of the black node; this activation then spreads to the three dark gray nodes connected to the initial node (the first tier or spreading activation), and from there the activation continues down all pathways connected to the first tier activated nodes to reach the light gray second tier of activated nodes. Anderson (1983a) suggests that the transmission of activation is bidirectional; as shown in **Figure 1**, nodes can rebound activation back upon nodes which are already activated (e.g., the light gray node outlined in a dashed black line rebounds activation received back to the initially activated node). The level of activation reached by each node begins to decrease as soon as the information contained in the node drops from the focus of attention (Anderson, 1983b) and continues to decrease with the passage of time (Collins and Loftus, 1975).

**Figure 1** also depicts the fanning of activation down parallel paths. Activation begins at the initially activated node and continues out along multiple parallel paths. Where an active concept node has links to multiple other nodes (these links are referred to as the fan of the concept), the activation spreads in parallel among these pathways. For example, the level of activation initially received at the source node (in black) splits simultaneously down the three pathways leading to the dark gray first tier activated nodes. Anderson (1983a) argues that nodes have a finite capacity for activation, and so the more paths a node is connected to, the less activation it is able to send down any one path (as the level of activation transmitted out along the path is a function of the amount of activation received minus the total number of paths connected to the node), and so the slower the recall process is. In essence, this means that where the fan effect occurs the amount of activation available for any one pathway decreases, and the time taken to retrieve information increases. The more facts that are linked to a given concept, the longer it takes to recall any one fact associated with that concept (Anderson and Reder, 1999).

Targets are recognized (or recalled) when a threshold level of activation has been reached (Anderson, 1983a). The overall amount of activation a given node receives predicts the amount of time it will take to accurately recall the information contained within that node (Anderson, 1983b). The level of activation that a node receives can be considered as a product of the strength of their associations. Nodes which are more closely or strongly related to the source of activation receive more activation than those which are further removed. In other words, as activation spreads throughout the network, its strength decreases. As Collins and Loftus (1975, p. 411) state “activation is like a signal from a source that is attenuated as it travels outward.” In this way, the level of activation of other nodes within the network varies in terms of their degree of association to the source nodes. The activation arriving from multiple sources at a single node will sum. As such, information contained within any given node is processed more quickly when multiple sources spread activation to the target node (Anderson and Pirolli, 1984). Ultimately the level of activation within a given area of the network predicts the speed and accuracy with which information within that area can be recalled (Anderson, 1983b). To illustrate, in **Figure 1** the information stored in nodes to the left of the vertical dotted line is more likely to be recalled quickly and accurately than the information stored in nodes on the right (all else being equal, the activation received by nodes on the left is greater than that received by those on the right). Individuals can also capitalize upon the gathering of activation within specific areas of a network by refocusing activation from the initial node to a more active subnode to enable faster a spread of activation (see Anderson, 1983b for discussion).

Within spreading activation models of memory there has been some debate around which factor ultimately predicts the time taken to recall a target item. It has previously been assumed that the time taken to recall an item is a function of the amount of time it takes activation to spread throughout the network (Ratcliff and McKoon, 1981). In contrast, Anderson (1983b) suggests that processing time can be explained as the time taken for activation to reach a peak (an asymptotic level of activation). This argument is based primarily on the findings of priming studies (see Anderson, 1983b for discussion), and is a key feature distinguishing Anderson’s (1983b) model of spreading activation from other spreading activation models. The strength of individual nodes and their associated links also contributes to understanding of how some nodes reach higher levels of activation sooner than others. One assumption of the fan effect described above is that as a node becomes active, each path from the concept node to its properties is equally activated. However, data suggests that this might not always be the case. As stated above, both Collins and Loftus (1975) and (Anderson, 1983a,b) argue that the strength of the relationship (and so the distance between) a node and the source of activation predicts how much activation that node is likely to receive. As a result, it can be assumed that not all concepts and links are of equal strength (Anderson, 1983a,b). For example, Anderson (1983a) suggests that activation is allocated among competing paths based upon their relative strength. He gives the example of slower response times for two-fan facts studied four times, when an alternative has been studied more frequently, and takes this as the basis for the argument that activation is allocated based upon the relative strength of each possible pathway (see Anderson, 1983a for further discussion).

Proponents of spreading activation theories of memory generally agree that individual nodes vary in strength. A number of explanations as to how this occurs have been put forward. For example, node strength may be predicted by frequency of exposure. When facts about concepts are studied and tested more frequently, the individual nodes containing these facts (and their associated memory traces) become stronger, resulting in faster, more accurate recall. This strengthening effect occurs even when practice sessions occur in quick succession (for further discussion of practice effects see Anderson, 1983b; Tuckey and Brewer, 2003). Anderson (1983b) argues that once formed traces are not lost, but their strength does decrease gradually over time. In this way, Schacter (1999) suggests that spreading activation theories of memory can go some way toward explaining what he refers to as ‘the sin of transience,’ or gradual forgetting over time. When not bolstered by the strengthening effects that retrieval attempts can have, the associated memory traces begin to gradually weaken, and so to become less accessible over time. On the other hand, Tuckey and Brewer (2003) argue that the strength of associative links is also in part determined by how schema-consistent or inconsistent the items encoded are. For example, aspects of an event that are schema consistent are more likely to be rehearsed and so are more likely to be strongly encoding than those that are schema inconsistent. This is supported by their finding that schema inconsistent information shows greater levels of decay than schema consistent information. Regardless of the reason for their strength, stronger nodes are also able to transmit and receive greater levels of activation, and thus allow more activation to gather in areas of the network containing stronger nodes (Anderson, 1983b). The implication of this for retrieval processes is that the most salient cues are the ones which are most likely to enable fast, accurate retrieval of information.

##### Spreading activation theory and self-generated cues

Spreading activation theories underpin the effectiveness of retrieval cues based upon a number of key properties. As has been previously discussed, a high-quality retrieval cue generally has a strong association with the target memory, whilst also being able to easily incorporate new related information as necessary. These associations should also be bidirectional, whereby the cue recalls the target information, and the target information recalls the cue (Bellezza and Hoyt, 1992). When the effectiveness of a retrieval cue is described in terms of these properties, then it is clear that the spreading activation theory of memory is of critical importance in explaining successful recall. We suggest that self-generated cues offer the opportunity to maximize the benefit of these properties, and briefly outline how this may be the case below.

It is well-established that recall of one item can prompt further recall of semantically related items (Collins and Loftus, 1975). This occurs through the spread of activation through the associative links of the memory network. When the associative links are stronger, then information is recalled faster and more accurately. For example, when recall of a target word is cued by a word more closely associated with the target then the target is recalled faster, than when the target is cued by a word situated further away in the network (Ratcliff and McKoon, 1981). The benefit of strongly associated semantic clusters has also been demonstrated through category clustering recall. In line with the spreading activation theory, if memory is indeed organized according to semantic similarity, then focusing on and recalling information by semantic cluster is likely to produce enough activation to cue associated items. When individuals are asked to make a second or third recall attempt using category clustering (i.e., attempting to recall further information one semantic category at a time, for example person details, action details, and so on), then recall improves without a cost to accuracy, compared to recall attempts using other established mnemonic techniques such as the change order mnemonic (Paulo et al., 2016). The prime benefit of this approach is that it is relatively intuitive; individuals often spontaneously encode, organize, and recall information in semantic clusters (see Paulo et al., 2016 for further discussion).

Although further research is needed to test these assumptions, we propose that self-generated cues represent a prime opportunity to capitalize upon the semantic organization of memory. In allowing individuals to define their own semantic clusters, we give individuals the opportunity to focus their recall attempts on clusters most compatible with their own encoding of the target material. Self-generated cues also present the opportunity to cue recall using strong associative links. In allowing individuals to generate their own cues we maximize the opportunity to trigger activation from the point most critical to the recall of the target material. For example, by allowing individuals to select their own cues we can capitalize upon the strongest associative links, and minimize the distance in the network between cue and target.

The importance of the bidirectionality of associative links becomes apparent when we consider ‘recognition failure’; where associative links do not have bidirectionality, then it is possible that a target memory will not be selected in a recognition context without the associated learned cue or context. Interestingly, this means that individuals may be able to recall details of the target memory given an associated concept that they are not able to provide in a recognition task (Tulving and Thomson, 1973; Wiseman and Tulving, 1976). Similarly, where a cue and target evoke each other with high frequency (e.g., tree cues oak, and vice versa) then the target is recalled more quickly when a cue is provided, than when a cue and target evoke each other with low frequency (e.g., cloth cueing orlon, or vice versa). Importantly, where the cue and target evoke each other with equal frequency then either word can be used to prompt recall of the other (i.e., it doesn’t matter which is presented as the cue, and which as the target). In contrast, where there is an imbalance in this strength of association, and so the cue evokes the target at a higher frequency than the inverse (as with seafood-shrimp; seafood evokes the word shrimp at a higher frequency than shrimp does seafood), then reaction time varies significantly dependent upon which word was used to cue which (Collins and Loftus, 1975). This demonstrates the importance of bidirectional relationships. We suggest that if self-generated cues do indeed offer the opportunity to minimize the distance between cue and target within the semantic network, then it is also plausible that they can contribute to maximizing the bidirectionality of associative links.

#### Encoding-Specificity Principle of Memory

Initially developed by Tulving and colleagues, the encoding-specificity principle of memory (or encoding-retrieval specificity) refers to the idea that retrieval cues are effective only to the extent that information within the memory cue is also contained within the target memory trace created at the time of encoding. As Tulving and Thomson (1973, p. 353) note “what is stored is determined by what is perceived and how it is encoded, and what is stored determines what retrieval cues are effective in providing access to what is stored.” Put another way, the encoding-specificity principle of memory takes as its core the idea that it is only possible to retrieve what has been stored in memory, and that the way this information has been encoded and stored governs the ways in which this information can be retrieved (Tulving and Thomson, 1973).

Tulving and Thomson (1973) agreed with the principles of memory outlined in spreading activation theories that: (a) information within memory is stored as a memory trace; (b) a memory trace is a collection of elements, features, or attributes of the encoded information; and (c) that an encoding phase is situated between the perception of an event, and the creation of a memory trace. However, they viewed retrieval as a selective process, relying on a complex interaction between encoded information and features of the retrieval environment (Tulving and Thomson, 1973). Tulving and Thomson (1973) argue that it is well-established that identical information encoded under different conditions can lead to differences in recall and recognition performance. Likewise, the information present at retrieval can greatly influence the recall and recognition of items stored under identical encoding conditions. These findings, as well as more general forgetting, can be explained through encoding-specificity in terms of the accessibility of information in memory; information may not be lost, so much as inaccessible given the cues available at the time of the recall attempt (Brown and Craik, 2000). Together, these ideas suggest that different cues might make different memory traces more accessible than others, which in turn raises the question of what constitutes an effective retrieval cue.

Tulving and Thomson (1973) argue that the spreading activation explanation of differences in recall performance as being caused by differing strengths of memory traces is of little practical value. Tulving and colleagues also suggest that the benefit of a strong cue-target association is likely to be lost if the cue is not also encoded alongside the target information (for further discussion see Tulving and Osler, 1968; Thomson and Tulving, 1970; Tulving and Thomson, 1973). If information is not salient at the time of encoding, then it will not act as an effective memory cue for the target, regardless of how central the cue might be to the target in general terms (Brown and Craik, 2000). In essence, this means that the match between features of recall and features of encoding is more important for a successful retrieval attempt than the strength of the association between the cue and the target information (Roediger and Guynn, 1996; Pansky et al., 2005).

A number of studies have demonstrated support for this concept. For example, across a series of three studies, Thomson and Tulving (1970) demonstrated that when weakly associated cues were encoded alongside target information, then strongly associated cues provided at recall (but not at encoding) did not facilitate retrieval of the target information. In addition, Higham (2002) found strongly associated retrieval cues not presented at encoding produced less correctly recalled information and more incorrect recall than weakly associated cues which had been previously presented at study. Furthermore, Rosenbluth-Mor (2001 cited in Pansky et al., 2005) found that weakly associated cues presented at both encoding and retrieval facilitated recall in comparison to a no cue control, whereas presenting a new (not seen at encoding) weakly associated cue at retrieval impaired performance in comparison to a no cue control. Taken together, these findings demonstrate that mismatch between encoding and retrieval cues impairs recall, rather than the more conventional view that increasing the match improves recall (Pansky et al., 2005). It is, however, important to note that this view is not universally shared by researchers. For example, research has shown that an encoding-retrieval mismatch has a more detrimental effect on those with high working memory capacity than those of low working memory capacity. It has been suggested that this effect is seen because individuals with high working memory capacity are more likely to encode information strategically, and to utilize these strategies at recall, and so experience a decline in performance when their planned strategies are disrupted (Unsworth et al., 2011). In addition, some researchers have found means of improving recall performance using strongly associated cues not presented at the time of encoding (see Higham, 2002, for discussion of this).

It is not the case that the encoding-specificity principle ignores the role that semantic relationships between cues and items to be recalled can play. Rather, this is seen as a part of the cognitive encoding environment. For example, when encoding a wordlist for later recall we can assume that information is encoded about the appearance of a given word in the present context. This might or might not include encoding information about the semantic relationships between wordlist items: if so then another item on the wordlist might constitute an effective retrieval cue, if not then this will not be the case (see Tulving and Thomson, 1973 for empirical support for these claims). In addition, where target words are encoded alongside cue words, there is often an assumption that these cues will reappear at test, and as such the cue word forms part of the context in which the target is encoded. This means that the target memory trace cannot always be readily accessed in a recognition context, where the memory cue provided consists solely of the target word itself without the associated encoding context. This is termed ‘recognition failure’ (see Wiseman and Tulving, 1976 for further discussion of recognition failure).

It should be noted that the encoding-specificity principle and the spreading activation theory are not necessarily mutually exclusive. Anderson (1983b) argues that the findings of encoding-specificity studies (such as Tulving and Thomson, 1973) can still be incorporated into a spreading activation framework. In particular, when a cue has multiple possible interpretations (e.g., the word ‘jam’ might be interpreted differently dependent upon whether it is presented alongside the associated word ‘raspberry’ or ‘traffic’), then the encoding context determines which interpretation is encoded (potentially alongside other cues from the encoding context itself). At retrieval, context can then be used to determine the appropriate interpretation to activate, and the activation spreads from this point out into the network. The probability of recall or recognition is therefore higher when the same interpretation is selected at both encoding and retrieval, thus allowing activation to spread directly from the node directly linked to the memory trace and reducing levels of activation sent down pathways linked to alternative interpretations.

##### Encoding-specificity and self-generated cues

As previously noted, the encoding-specificity principle of memory and spreading activation theory are not mutually exclusive. Context can be used to activate appropriate concepts within memory (Anderson, 1983b), and facilitate the spread of activation through a memory network (Hershkowitz et al., 2002). Research around the generation of cues for the self versus another individual suggests that self-generated cues contain more idiosyncratic episodic details than cues generated by, or for use by, others. The latter tend to contain more generic, semantic details (Mäntylä, 1986; Mäntylä and Nilsson, 1988). Interestingly, cues generated by older adults to cue their own memory also tend to show this same generic focus (Mäntylä and Bäckman, 1990). In addition, when learners recall an incorrect target in response to a self-generated cue this seems to be driven by a strong associative relationship between the cue and the incorrect response (Tullis, 2013). Taken together, these findings suggest that spreading activation can be considered as a ‘back-up’ route in cue generation, seemingly forming a default option when cognitive resources are low, or when recall via a more efficient means (such as encoding-specificity or cue distinctiveness) has failed. In this sense, spreading activation theory can essentially be viewed as the foundation upon which effective retrieval cues, whether generated by the self or another, can be built, with encoding-specificity and cue distinctiveness providing an additional benefit beyond this default route.

The encoding-specificity principle of memory suggests that good quality retrieval cues have a high level of overlap between encoding and retrieval. This allows cues generated at encoding to be reproduced at retrieval reliably and consistently. These qualities, combined with the benefit of semantic clustering, make for highly effective retrieval techniques. For example, while the category clustering recall technique previously outlined allows recall to be cued using strongly associated semantic clusters, this technique also provides the additional benefit of framing recall in an encoding compatible manner. The same benefit is provided by self-generated cues; indeed, we would suggest that this benefit is magnified in the case of self-generated cues. According to the principle of encoding-retrieval specificity, effective cueing relies on a knowledge of the most salient aspects of information to be recalled. If this is the case then it follows logically that the best cues are generated by the self to guide recall, rather than by an other.

#### Cue Distinctiveness

Overall, the idea that the same material may be encoded differently in a different cognitive context, resulting in different routes through which to access the information, lies at the heart of the encoding-specificity principle of memory. Yet, Tulving and Thomson (1973) also highlight the influence of other, somewhat indefinable factors. They demonstrate that an additional factor is likely to operate alongside the properties of an encoded item, and that this unknown factor further impacts upon the chance of successful retrieval. As Nairne (2002) states, even when we ensure a *nominal* match between encoding and retrieval (e.g., through use of identical cues), this does not guarantee a *functional* match between the cue and the memory trace for the target item. Therefore, despite the widely accepted beliefs that once encoding has been completed it is the match between encoding and retrieval conditions that is the primary predictor of memory performance, data from memory studies (see Nairne, 2002) suggest that there must be other factors also at play. One candidate which may help to explain the differences in recall performance not captured by encoding-specificity, is cue distinctiveness^1^.

Nairne (2002, p. 390) considers the process of remembering to be “an active process of discrimination” during which we use retrieval cues to guide us toward viable retrieval candidates. He argues that although the encoding-specificity principle of memory is of some practical value, its theoretical relevance is limited. The rationale behind this claim is that the relationship between encoding and retrieval is correlational rather than causal. Instead Nairne (2002) argues that cue distinctiveness has a stronger influence on retrieval. Increasing the overlap between encoding and retrieval benefits recall through increasing the probability that distinctive features unique to the target will be utilized. He is not alone in this belief; it has been suggested that a key property of an effective retrieval cue is discriminability (Bellezza and Hoyt, 1992). Retrieval cues which are distinct from each other are more likely to prompt the recall of target information, and more likely to result in the recall of verbatim, rather than gist-based information (Anderson, 1983a; Anderson and Reder, 1999; Tuckey and Brewer, 2003). Cue distinctiveness is based upon similar principles.

Cue distinctiveness (or an absence of cue overload) refers to whether a cue is uniquely associated with a target memory. If a cue is linked to multiple memory traces (and so is ‘overloaded’), then it becomes more difficult for that cue to activate the current target trace. This clearly will reduce the effectiveness of the cue in facilitating recall of the target information (Watkins and Watkins, 1975). In other words, a retrieval cue is useful only to the extent that it provides diagnostic information about the occurrence of a target item (Pansky et al., 2005). Cue distinctiveness is also entwined with the encoding process. Encoding information in ways that lead to a more precise memory trace, and in doing so separating one encoding experience from others contained within memory, facilitates recall. Distinctiveness is critical to this process (see Schmidt, 1991, for a review of the distinctiveness literature). When unique elements of an event (those which do not overlap with other events) are encoded, then these elements form a unique identifier for the target event, and so increase the likelihood that it can be discriminated from other events stored in memory. Where this distinct element is available at retrieval then the unique cue reinstates the original memory trace, provided that the context (of the distinctive element) is the same (Hunt and Smith, 1996).

Most researchers currently favor a two-factor account, which accepts that both encoding-retrieval match (encoding-specificity) and cue overload (or cue distinctiveness) combine to influence memory performance. However, Nairne (2002) argues that this approach impedes our ability to make practical predictions about memory performance. He gives an example of trying to recall a target event (E_1_) from a series of events (E_2_, E_3_, and so on). If a participant is cued with an event feature unique to the target event (feature X_1_), then this is likely to facilitate recall. However, if the feature used as a cue was present for events one, two, and three (E_1_, E_2_, E_3_), then this cue (feature X_2_) loses its diagnostic value, making it more difficult to discriminate the target event memory from other competing event memories. In this case, we can reasonably expect recall performance to decline. In short, memory performance is equal to the match between cue (X_1_) and target (E_1_) and declines as the number of items associated with cue (X_1_) increases (Nairne, 2002). The critical aspect of the cue distinctiveness principle then is that cue-target match is *necessary* but not *sufficient* for accurate retrieval. Nairne (2002) and other advocates of the benefit of cue distinctiveness (e.g., Moscovitch and Craik, 1976) accept that retrieval cues are effective only if they match the memory trace of the target item (as in the encoding-specificity principle of memory), but suggest that diagnostic cues, which specify a single target item and exclude others, are key in predicting recall performance. In other words, if a retrieval cue is specific to the encoded event, then this is more likely to result in accurate recall than a more generic cue, and it is this diagnostic value that is key (Nairne, 2002; Goh and Lu, 2012).

Several studies have shown support for cue distinctiveness as a predictor of recall performance. For example, Moscovitch and Craik (1976, Experiments 2 and 3) manipulated the number of targets paired with a cue, and the similarity of this cue to others encoded. Participants encoded questions as cues alongside target words, and were then asked to recall the target words given the question cue. When cues were shared among a set of 10 targets, recall performance was lower than when each target was prompted by a distinct cue question. This is consistent with other research (e.g., Watkins and Watkins, 1975) and with well-documented effects such as the list length effect. However, Moscovitch and Craik’s findings suggest that this effect was not universal across all stimuli (for example semantically encoded words, or items associated with a positive response to the cue question). In addition, they noted that recall of rhyme-encoded words showed little decline in response to the shared cue manipulation. They argue that this suggests that there are ‘levels’ of distinctiveness, and that surface level distinctiveness is of little importance in comparison to more meaningful forms of distinctiveness. In order to test this hypothesis, Goh and Lu (2012), manipulated both encoding-retrieval match and the degree of cue overload in a 2 (overload: high, low) × 2 (encoding-retrieval match: high, low) design. In each condition participants learned a list of word pairs and were later tested on these pairs in a cued recall task. In high encoding-retrieval match conditions participants were provided with the originally encoded cue word, alongside a second cue of the semantic category the target word belonged to. In low encoding-retrieval match conditions, only the originally encoded cue was provided. To manipulate cue overload, Goh and Lu (2012) ensured that the semantic category cue provided at test applied to several (in some cases all) of the words learned at encoding (high cue overload) or was unique to the target word (low cue overload). Goh and Lu’s (2012) results suggest that high encoding-retrieval match does not necessarily facilitate recall, showing instead that high encoding-retrieval match improves performance only when cue overload is low (see Brandimonte and Passolunghi, 1994, for similar support of cue-distinctiveness in a prospective memory task).

##### Cue distinctiveness and self-generated cues

The principles of encoding-specificity and cue distinctiveness can be difficult to disentangle in terms of their contribution to the effectiveness of retrieval cues, and of self-generated cues in particular. It is clear, however, that cue distinctiveness adds to the effectiveness of cues with a high degree of encoding-retrieval overlap. For example, while the effectiveness of a cue which has a high level of overlap with the target, and contains idiosyncratic details about the encoding context can be understood in terms of encoding-specificity, maintaining this advantage can be seen as a product of cue distinctiveness. In other words, the best retrieval cues are those which emphasize distinctive aspects of the target, resulting in increased consistency with which targets are produced in response to cues over a longer retention interval. Where this consistency is lost, we see increased encoding variability, and poorer memory performance over time (Watkins and Watkins, 1975; Mäntylä and Bäckman, 1990; Anderson and Reder, 1999). Asking learners to focus specifically on distinctive aspects of the to-be-recalled information during encoding results in self-generated cues which maximize distinctiveness in a way that is unaffected by changes in context (reduced levels of encoding-retrieval match), and in turn ensures that levels of performance are maintained over time (Mäntylä and Nilsson, 1988). In addition, the idiosyncratic nature of self-generated cues means that one individual’s cues that are given to another individual at test would be unlikely to benefit their performance, even if the same information had been presented at encoding. This additional benefit of cue distinctiveness beyond merely cue-target overlap demonstrates the separate qualities that cue distinctiveness and encoding-specificity bring to effective self-generated cues. Cue distinctiveness is naturally maximized where cues are self-generated. Where individuals generate cues for use by others, they tend to revert back to more general, semantic, gist-based aspects of the target information, rather than the more specific idiosyncratic episodic details incorporated into self-generated cues. In this way, self-generated retrieval cues capitalize upon cue distinctiveness, and so maximize the effectiveness of the cue (Mäntylä, 1986; Hunt and Smith, 1996).

## Conclusion

Successful recall of information from memory is often dependent upon the provision of retrieval cues. Retrieval cues might form part of the retrieval context, and can be self or other-generated (Pansky et al., 2005). In line with the spreading activation theory of memory, and the principles of encoding-specificity, and cue distinctiveness, effective retrieval cues are often strongly associated with the target item, have a strong cue-target overlap, and differentiate between different items stored within memory (Bellezza and Hoyt, 1992; Tullis and Benjamin, 2015a). Based upon the literature discussed, we argue that if self-generated cues are taken to be cues containing details salient to the individual, and actively generated by the individual themselves, which serve to facilitate more complete retrieval of a target memory, and as such represent the critical properties of the target memory, then it follows logically that self-generated retrieval cues represent our best opportunity to capitalize upon these three principles of memory. In particular, it is in relation to the principle of cue distinctiveness that self-generated cues offer an advantage over other mnemonic techniques (e.g., Mental Reinstatement of Context). While other-generated cues rely heavily upon more general, semantic, gist-based aspects of the target information, self-generated cues are able to incorporate more specific idiosyncratic episodic details to maximize the diagnostic value of a cue (Nairne, 2002). This important when it is considered that the benefits of context-based cues become more apparent only when more effective cues are unavailable. In other words, the benefit of context-based mnemonic approaches can be overshadowed if individuals are able to provide their own cues (Pansky et al., 2005).

Overall, the literature discussed suggests that self-generated cues represent an effective and viable mnemonic technique which can aid recall in a variety of settings. The high level of compatibility of self-generated cues with individual requirements and abilities means they do not require complex training or regular practice to be used effectively. As a result, we suggest that self-generated cues represent a promising development in episodic memory domains. Throughout the preceding discussion we have speculated on the effectiveness of self-generated cues, however, further research is needed to establish the extent of the contribution self-generated cues are able to make to the field. In particular, future research should seek to replicate existing findings on the benefit of self-generated cues, especially in comparison to other mnemonic techniques such as Mental Reinstatement of Context, or category clustering techniques. Future research is also needed to extend current knowledge of the most effective means of self-generating retrieval cues. For example, through establishing the qualities of an effective cue generation technique, and by contrasting existing methods of cue generation. Future research should also seek to establish the boundary conditions of effective self-generated cues. For instance, under what conditions are self-generated cues most effective, or what impact does varying the delay between encoding, cue generation, and recall have upon retrieval. It may also be of interest to investigate whether use of self-generated cues improve item memory, but reduce memory for context as has been shown with the generation effect (Mulligan, 2004; Mulligan et al., 2006). It is also important to establish the potential implications of use of self-generated cues in a variety of settings, for example in eyewitness testimony contexts, educational settings, and during collaborative learning and recall. Throughout this article we have also speculated on how spreading activation theories, the encoding-specificity principle of memory, and cue distinctiveness each contribute to the effectiveness of self-generated cues. While we acknowledge that these principles are often strongly intertwined, we believe that it would be beneficial for future research to address which of the mechanisms outlined contributes most strongly to the success of self-generated cue techniques.

## Author Contributions

RW and FG devised the concept of the manuscript. RW drafted the work, and FG critically revised it. The final manuscript was approved by RW and FG.

## Conflict of Interest Statement

The authors declare that the research was conducted in the absence of any commercial or financial relationships that could be construed as a potential conflict of interest.

## Footnotes

1 It should be noted that some researchers distinguish between the terms ‘unique’ and ‘distinctive’ (see Mäntylä and Nilsson, 1983 for discussion of this). While we agree with Mäntylä and Nilsson (1983) that a careful conceptual analysis of these terms is needed, this is beyond the scope of this paper. Therefore, throughout this manuscript we use the terms unique and distinctive interchangeably to describe a retrieval cue which recalls one particular memory at the exclusion of others, and as such can be considered to have diagnostic value.

## References

[B1] Anderson J. R. (1983a). Retrieval of information from long-term memory. *Science* 220 25–30. 10.1126/science.6828877 6828877

[B2] Anderson J. R. (1983b). A spreading activation theory of memory. *J. Verbal Learn. Verbal Behav.* 22 261–295. 10.1016/S0022-5371(83)90201-3

[B3] Anderson J. R. Pirolli P. L. (1984). Spread of activation. *J. Exp. Psychol.* 10 791–798. 10.1037/0278-7393.10.4.791

[B4] Anderson J. R. Reder L. M. (1999). The fan effect: new results and new theories. *J. Exp. Psychol.* 128 186–197. 10.1037/0096-3445.128.2.186 17196744

[B5] Andersson J. Ronnberg J. (1997). Cued memory collaboration?: effects of friendship and type of retrieval cue. *Eur. J. Cogn. Psychol.* 9 273–288. 10.1080/713752558

[B6] Bellezza F. S. Hoyt S. K. (1992). The self-reference effect and mental cueing. *Soc. Cogn.* 10 51–78. 10.1521/soco.1992.10.1.51

[B7] Brainerd C. J. Reyna V. F. Brandse E. (1995). Are children’s false memories more persistent than their true memories? *Psychol. Sci.* 6 359–364. 10.1111/j.1467-9280.1995.tb00526.x

[B8] Brandimonte M. A. Passolunghi M. C. (1994). The effect of cue-familiarity, cue-distinctiveness, and retention interval on prospective remembering. *Q. J. Exp. Psychol. Hum. Exp. Psychol.* 47 565–587. 10.1080/14640749408401128 7938668

[B9] Brown C. Lloyd-Jones T. J. Robinson M. (2008). Eliciting person descriptions from eyewitnesses: a survey of police perceptions of eyewitness performance and reported use of interview techniques. *Eur. J. Cogn. Psychol.* 20 529–560. 10.1080/09541440701728474

[B10] Brown S. Craik F. I. M. (2000). “Encoding and retrieval of information,” in *The Oxford Handbook of Memory* eds Tulving E. Craik F. I. M. (Oxford: Oxford University Press) 93–107.

[B11] Collins A. M. Loftus E. F. (1975). A spreading-activation theory of semantic processing. *Psychol. Rev.* 82 407–428. 10.1037/0033-295X.82.6.407

[B12] Conway M. A. (2001). Sensory-perceptual episodic memory and its context: autobiographical memory. *Philos. Trans. R. Soc. B Biol. Sci.* 356 1375–1384. 10.1098/rstb.2001.0940 11571029 PMC1088521

[B13] Conway M. A. Pleydell-Pearce C. W. (2000). The construction of autobiographical memories in the self-memory system. *Psychol. Rev.* 107 261–288. 10.1037//0033-295X 10789197

[B14] Crestani F. (1997). Application of spreading activation techniques in information retrieval. *Artif. Intellig. Rev.* 11 453–482. 10.1023/A:1006569829653

[B15] Dando C. J. Wilcock R. Behnkle C. Milne R. (2011). Modifying the cognitive interview: countenancing forensic application by enhancing practicability. *Psychol. Crime Law* 17 491–511. 10.1080/10683160903334212

[B16] Dando C. J. Wilcock R. Milne R. Henry L. A. (2009). A modified cognitive interview procedure for frontline police investigators. *Appl. Cogn. Psychol.* 23 698–716. 10.1002/acp.1501

[B17] Derwinger A. Neely A. S. Bäckman L. (2005). Design your own memory strategies! Self-generated strategy training versus mnemonic training in old age: an 8-month follow-up. *Neuropsychol. Rehabil.* 15 37–54. 10.1080/09602010343000336 16353852

[B18] Derwinger A. Neely A. S. Persson M. Hill R. D. Bäckman L. (2003). Remembering numbers in old age: mnemonic training versus self-generated strategy training. *Aging Neuropsychol. Cogn.* 10 202–214. 10.1076/anec.10.3.202.16452

[B19] Fisher R. P. Geiselman R. E. Holland H. L. MacKinnon D. P. (1984). Hypnotic and cognitive interviews to enhance the memory of eyewitnesses to crime. *Int. J. Invest. Foren. Hypn.* 7 28–31. 9549881

[B20] Fivush R. (2008). Remembering and reminiscing: How individual lives are constructed in family narratives. *Mem. Stud.* 1 49–58. 10.1177/1750698007083888 22044305

[B21] Geiselman R. E. Fisher R. P. MacKinnon D. P. Holland H. L. (1985). Eyewitness memory enhancement in the police interview: cognitive retrieval mnemonics versus hypnosis. *J. Appl. Psychol.* 70 401–412. 10.1037/0021-9010.70.2.401 3997740

[B22] Geiselman R. E. Fisher R. P. MacKinnon D. P. Holland H. L. (1986). Enhancement of eyewitness memory with the cognitive interview. *Am. J. Psychol.* 99 385–401. 10.2307/1422492 3997740

[B23] Goh W. D. Lu S. H. X. (2012). Testing the myth of the encoding–retrieval match. *Mem. Cogn.* 40 28–39. 10.3758/s13421-011-0133-9 21830162

[B24] Greenwald A. G. Banaji M. R. (1989). The self as a memory system: Powerful, but ordinary. *J. Pers. Soc. Psychol. Pers. Soc. Psychol.* 57 41–54. 10.1037/0022-3514.57.1.41 23882210

[B25] Gutchess A. H. Kensinger E. A. Yoon C. Schacter D. L. (2007). Ageing and the self-reference effect in memory. *Memory* 15 822–837. 10.1080/09658210701701394 18033620

[B26] Harris C. B. Rasmussen A. S. Berntsen D. (2014). The functions of autobiographical memory: an integrative approach. *Memory* 22 559–581. 10.1080/09658211.2013.806555 23808866

[B27] Hershkowitz I. Orbach Y. Lamb M. E. Sternberg K. J. Horowitz D. (2002). A comparison of mental and physical context reinstatement in forensic interviews with alleged victims of sexual abuse. *Appl. Cogn. Psychol.* 16 429–441. 10.1002/acp.804

[B28] Higham P. A. (2002). Strong cues are not necessarily weak: thomson and Tulving (1970) and the encoding specificity principle revisited. *Mem. Cogn.* 30 67–80. 10.3758/BF03195266 11958356

[B29] Hope L. Mullis R. Gabbert F. (2013). Who? What? When? Using a timeline technique to facilitate recall of a complex event. *J. Appl. Res. Mem. Cogn.* 2 20–24. 10.1016/j.jarmac.2013.01.002

[B30] Hunt R. R. Smith R. E. (1996). Accessing the particular from the general: the power of distinctiveness in the context of organization. *Mem. Cogn.* 24 217–225. 10.3758/BF03200882 8881324

[B31] Kebbell M. R. Milne R. Wagstaff G. (1999). The cognitive interview: a survey of its forensic effectiveness. *Psychol. Crime Law* 5 101–115. 10.1080/10683169908414996 16844797

[B32] Krafka C. Penrod S. D. (1985). Reinstatement of context in a field experiment on eyewitness identification. *J. Pers. Soc. Psychol. Pers. Soc. Psychol.* 49 58–69. 10.1037/0022-3514.49.1.58

[B33] Laffan A. J. Metzler-Baddeley C. Walker I. Jones R. W. (2010). Making errorless learning more active: self-generation in an error free learning context is superior to standard errorless learning of face–name associations in people with Alzheimer’s disease. *Neuropsychol. Rehabil.* 20 197–211. 10.1080/09602010903202432 19787546

[B34] Leins D. A. Fisher R. P. Pludwinski L. Rivard J. Robertson B. (2014). Interview protocols to facilitate human intelligence sources’ recollections of meetings. *Appl. Cogn. Psychol.* 28 926–935. 10.1002/acp.3041

[B35] Mäntylä T. (1986). Optimizing cue effectiveness: recall of 500 and 600 incidentally learned words. *J. Exp. Psychol. Learn. Mem. Cogn.* 12 66–71. 10.1037/0278-7393.12.1.66

[B36] Mäntylä T. Bäckman L. (1990). Encoding variability and age-related retrieval failures. *Psychol. Aging* 5 545–550. 10.1037/0882-7974.5.4.545 2278678

[B37] Mäntylä T. Nilsson L.-G. (1983). Are my cues better than your cues? *Scand. J. Psychol.* 24 303–312. 10.1111/j.1467-9450.1983.tb00504.x

[B38] Mäntylä T. Nilsson L.-G. (1988). Cue distinctiveness and forgetting: effectiveness of self-generated retrieval cues in delayed recall. *J. Exp. Psychol. Learn. Mem. Cogn.* 14 502–509. 10.1037/0278-7393.14.3.502

[B39] Memon A. Meissner C. A. Fraser J. (2010). The cognitive interview: a meta-analytic review and study space analysis of the past 25 years. *Psychol. Public Policy Law* 16 340–372. 10.1037/a0020518

[B40] Moscovitch M. Craik F. I. M. (1976). Depth of processing, retrieval cues, and uniqueness of encoding as factors in recall. *J. Verbal Learn. Verbal Behav.* 15 447–458. 10.1016/S0022-5371(76)90040-2

[B41] Mulligan N. W. (2004). Generation and memory for contextual detail. *J. Exp. Psychol. Learn. Mem. Cogn.* 30 838–855. 10.1037/0278-7393.30.4.838 15238028

[B42] Mulligan N. W. Lozito J. P. Rosner Z. A. (2006). Generation and context memory. *J. Exp. Psychol. Learn. Mem. Cogn.* 32 836–846. 10.1037/0278-7393.32.4.836 16822151

[B43] Nairne J. S. (2002). The myth of the encoding-retrieval match. *Memory* 10 389–395. 10.1080/09658210244000216 12396651

[B44] Pansky A. Koriat A. Goldsmith M. (2005). “Eyewitness recall and testimony,” in *Psychology and Law an Empirical Perspective* eds Brewer N. Williams K. D. (New York, NY: Guilford Press) 93–150.

[B45] Paulo R. M. Albuquerque P. B. Bull R. (2016). Improving the enhanced cognitive interview with a new interview strategy: category clustering recall. *Appl. Cogn. Psychol.* 30 775–784. 10.1002/acp.3253

[B46] Quillian M. R. (1962). A revised design for an understanding machine. *Mech. Trans.* 7 17–29.

[B47] Quillian M. R. (1967). Word concepts: a theory and simulation of some basic semantic capabilities. *Behav. Sci.* 12 410–430. 10.1002/bs.3830120511 6059773

[B48] Ratcliff R. McKoon G. (1981). Does activation really spread? *Psychol. Rev.* 88 454–462. 10.1037/0033-295X.88.5.454

[B49] Roediger H. L. Guynn M. J. (1996). “Retrieval processes,” in *Human Memory* eds Bjork E. L. Bjork R. A. (San Diego, CA: Academic Press) 197–236.

[B50] Sauzéon H. Rodrigues J. Corsini M.-M. N’Kaoua B. (2013). Age-related differences according to the associative deficit and the environmental support hypotheses: an application of the formal charm associative memory model. *Exp. Aging Res.* 39 275–304. 10.1080/0361073X.2013.779192 23607398

[B51] Schacter D. L. (1999). The seven sins of memory. *Am. Psychol.* 54 182–203. 10.1037/0003-066X.54.3.182 10199218

[B52] Schmidt S. R. (1991). Can we have a distinctive theory of memory? *Mem. Cogn.* 19 523–542. 10.3758/BF03197149 1758300

[B53] Slamecka N. J. Graf P. (1978). The generation effect: delineation of a phenomenon. *J. Exp. Psychol.* 4 592–604. 10.3758/BF03198475

[B54] Smith S. M. (1979). Remembering in and out of context. *J. Exp. Psychol. Hum. Learn. Mem.* 5 460–471. 10.1037/0278-7393.5.5.460

[B55] Symons C. S. Johnson B. T. (1997). The self-reference effect in memory: a meta-analysis. *Psychol. Bull.* 121 371–394. 10.1037/0033-2909.121.3.371 9136641

[B56] Thomson D. M. Tulving E. (1970). Associative encoding and retrieval: weak and strong cues. *J. Exp. Psychol.* 86 255–262. 10.1037/h0029997

[B57] Tuckey M. R. Brewer N. (2003). The influence of schemas, stimulus ambiguity, and interview schedule on eyewitness memory over time. *J. Exp. Psychol. Appl.* 9 101–118. 10.1037/1076-898X.9.2.101 12877270

[B58] Tullis J. G. (2013). *Cue Generation: How learners flexibly support future retrieval.* Champaign, IL: University of Illinois. 10.3758/s13421-015-0517-3 25777138

[B59] Tullis J. G. Benjamin A. S. (2015a). Cue generation: how learners flexibly support future retrieval. *Mem. Cogn.* 43 922–938. 10.3758/s13421-015-0517-3 25777138

[B60] Tullis J. G. Benjamin A. S. (2015b). Cueing others’ memories. *Mem. Cogn.* 43 634–646. 10.3758/s13421-014-0478-y 25377508

[B61] Tulving E. (1972). Episodic and semantic memory. *Organ. Mem.* 1 381–403.

[B62] Tulving E. (1974). Cue dependent forgetting. *Am. Sci.* 62 74–82.

[B63] Tulving E. (1985). Memory and consciousness. *Can. Psychol.* 26 1–12. 10.1037/h0080017

[B64] Tulving E. (2001). Episodic memory and common sense: how far apart? *Philos. Trans. R. Soc. B Biol. Sci.* 356 1505–1515. 10.1098/rstb.2001.0937 11571040 PMC1088532

[B65] Tulving E. (2002). Episodic memory: from mind to brain. *Annu. Rev. Psychol.* 53 1–25. 10.1146/annurev.psych.53.100901.135114 11752477

[B66] Tulving E. Osler S. (1968). Effectiveness of retrieval cues in memory for words. *J. Exp. Psychol.* 77 593–601. 10.1037/h0026069 5672271

[B67] Tulving E. Thomson D. M. (1973). Encoding specificity and retrieval processes in episodic memory. *Psychol. Rev.* 80 352–373. 10.1037/h0020071

[B68] Turk D. J. Gillespie-Smith K. Krigolson O. E. Havard C. Conway M. A. Cunningham S. J. (2015). Selfish learning: the impact of self-referential encoding on children’s literacy attainment. *Learn. Instr.* 40 54–60. 10.1016/j.learninstruc.2015.08.001

[B69] Unsworth N. Brewer G. A. Spillers G. J. (2011). Variation in working memory capacity and episodic memory: examining the importance of encoding specificity. *Psychon. Bull. Rev.* 18 1113–1118. 10.3758/s13423-011-0165-y 21912997

[B70] van Dam G. Brinkerink-Carlier M. Kok I. (1987). The effects of self-generated cues on recall of the paragraphs of a text. *J. Gen. Psychol.* 114 135–146. 10.1080/00221309.1987.9711064

[B71] Watkins O. C. Watkins M. J. (1975). Buildup of proactive inhibition as a cue-overload effect. *J. Exp. Psychol. Hum. Learn. Mem.* 1 442–452. 10.1037/0278-7393.1.4.442

[B72] Wiseman S. Tulving E. (1976). Encoding specificity: relation between recall superiority and recognition failure. *J. Exp. Psychol. Hum. Learn. Mem.* 2 349–361. 10.1037/0278-7393.2.4.349

